# Seeking healthcare services post-stroke: a qualitative descriptive study exploring family caregiver and stroke survivor perspectives in an asian setting

**DOI:** 10.1186/s12883-021-02463-7

**Published:** 2021-11-05

**Authors:** Shilpa Tyagi, Nan Luo, Chuen Seng Tan, Kelvin Bryan Tan, Boon Yeow Tan, Edward Menon, N. Venketasubramanian, Wei Chin Loh, Shu Hui Fan, Kenneth Lam Thuan Yang, Audrey Swee Ling Chan, Aysha Farwin, Zunairah Binti Lukman, Gerald Choon-Huat Koh

**Affiliations:** 1 Saw Swee Hock School of Public Health, National University of Singapore, 12 Science Drive 2, #10-01, 117549 Singapore, Singapore; 2 Policy Research & Economics Office, Ministry of Health, Singapore, Singapore; 3 St. Luke’s Hospital, Singapore, Singapore; 4 St. Andrew’s Community Hospital, Singapore, Singapore; 5 Raffles Neuroscience Centre, Raffles Hospital, Singapore, Singapore; 6 Singapore National Stroke Association, Singapore, Singapore

**Keywords:** Stroke, Caregiving, Family caregivers, Healthcare utilization, Qualitative research

## Abstract

**Aim:**

Exploration of the healthcare journey post-stroke is incomplete without acknowledging the crucial role of family caregivers. With limited literature documenting the role of caregivers in the healthcare journey post-stroke, we aimed to describe the healthcare experiences of family caregivers and stroke survivors across different caregiver identities in Singapore.

**Methods:**

We conducted a qualitative descriptive study involving semi-structured interviews with transcripts analysed using thematic analysis. 26 stroke survivors and 35 family caregivers purposively sampled from multiple settings.

**Results:**

Findings were summarized into seeking care and experience of healthcare encounters. Seeking care comprised of the following themes: factors influencing seeking care, decision to seek care and role of caregiver in seeking care. Experience of healthcare encounters comprised of the following themes: service around the patient, service with care and role of caregiver in healthcare encounters.

**Conclusion:**

Multi-dimensional role of caregivers in healthcare experience emerged as a major finding. Unique to our Asian context, as per the participants’ accounts, family caregivers seemed to be central in healthcare decision-making for stroke survivors, with adult-child caregivers commonly reported being engaged in collaborative decision-making. While spousal caregivers preferred a relational healthcare experience, adult-child caregivers preferred a transactional one. Practical implications include equipping caregivers with skillset to make healthcare decisions, provision of supportive decision-making environment for caregivers and reinforcing communication aspects in the medical, nursing and allied healthcare curriculum to improve healthcare experience.

**Supplementary Information:**

The online version contains supplementary material available at 10.1186/s12883-021-02463-7.

## Introduction

Exploration of the healthcare journey post-stroke is incomplete without acknowledging the crucial role of family caregivers in providing unfading support and care for their loved ones. After surviving acute stroke event, stroke survivors face challenges on multiple fronts with significant physical, emotional, and cognitive impairments, with up to 74 % requiring someone, often a family member, to aid with activities of daily living (ADL) [1], and more than 50 % requiring full assistance in some instrumental activities of daily living (IADL) domains [2]. These multidimensional needs post-stroke necessitate the presence of a partner accompanying the stroke survivor on a post-stroke recovery trajectory. With almost half of stroke survivors discharged home after the acute event [3], often a family member or a friend partners with the stroke survivor and takes up the caregiving role, extending both in community and healthcare settings.

Family caregivers, also known as informal caregivers, have been previously defined as “*relatives, partners, friends, or neighbors who provide help because of a personal relationship and who provide personal and medically oriented care*” [4]. They are unpaid and often without any formal training [4]. Caregiving entails multiple tasks of varying intensity, complexity and proficiency, especially for more medically inclined tasks. Among caregivers involved in complex chronic care, not only do they help with ADL, about 46 % engage in medical and nursing tasks [5]. About 57 % of caregivers express having no choice in engaging in these clinical tasks, considering this as their obligation [5]. The prevalence of informal caregiving ranges from 24 to 51 % with the caregiving duration at times amounting to 0.5 to 1 full-time equivalent of help where 1 full-time equivalent refers to an individual’s 1 full workday [6, 7]. Despite caregiving being valued financially at upto $14.2 billion annually in the United States [8], family caregivers are often unacknowledged and unsupported by current policy and practice guidelines [9]. While the caregiving experience of caregivers of stroke survivors has been well documented in the literature [10–13], there has been limited exploration of healthcare experiences of caregivers of stroke survivors along with describing their role in this healthcare setting. It is understandably challenging to describe the caregiver role in the healthcare journey of the care recipient considering the inherent complexity and context dependent nature [14]. While there is some literature describing the role of caregivers in post-stroke rehabilitation, highlighting them as stakeholders, legitimate clients and healthcare navigators [15], there is an existing gap in literature on exploring the role of caregivers in the healthcare experience of stroke survivors across the whole care continuum and multiple care settings. Our study would be addressing this gap.

We need to unpack the intersection of caregiving and healthcare experience to further establish caregivers’ position within the healthcare system, which would guide the provision of tailored support resulting in favourable stroke survivor-caregiver dyadic outcomes. From a quantitative perspective, researchers have examined the role of caregivers in health services utilization post-stroke, with studies reporting caregiver availability [16], co-residing status [17] and social support [18, 19] being associated with reduced risk of hospitalizations. Caregiver identity was reported to be significantly associated with the use of acute care services, specifically stroke survivors having an adult-child caregiver had almost three times greater rate of hospitalizations over the late post-stroke period as compared to spousal caregiver [19]. While these quantitative findings establish caregiver’s role in the post-stroke health services utilization, there still exists a gap in literature related to qualitative exploration of such healthcare experiences to provide a more comprehensive account with potentially actionable information. Our study would be addressing this gap across different caregiver identities.

On the qualitative front, a study explored stroke survivors’ and their caregivers’ perspectives on factors associated with seeking acute care services post-stroke concluding rehospitalizations to be multi-factorial [20]. A meta-ethnographic synthesis involving 168 stroke survivors and 328 informal caregivers reported perceived marginalization of the dyads by the primary and outpatient services with limited information availability and access, discontinuity of care and fluidity of stroke related needs [21]. Not only are the qualitative studies exploring the healthcare experiences of caregivers and stroke survivors scant, but there are also some evident gaps in this existing literature. Firstly, none of the included studies were from Asian settings where the caregiving experience may be socio-culturally different from Western settings. Secondly, the healthcare system attributes may vary across different settings potentially influencing the healthcare experiences. Lastly, none of these studies explicitly examined the differences in healthcare experiences across different caregiver identities (e.g., spouse, adult-child, sibling or others as caregivers). Considering the caregiving experience has been reported to vary across different caregiver identities [22–24], the healthcare experience may also vary across different caregiver identities. Addressing these gaps in literature, our study aimed to describe the healthcare experiences of family caregivers and stroke survivors in an Asian setting. Additionally, we aimed to explore the differences in healthcare experiences of caregivers and stroke survivors across different caregiver identities (i.e., spouse, adult-child, etc.).

## Methods

We adopted a qualitative descriptive study design involving semi-structured interviews with stroke survivors and family caregivers [25]. Our study was approved by the National University of Singapore’s Institutional Review Board (NUS-IRB Ref No: S-18-204). Written informed consent was obtained from both the patients and the caregivers in their preferred language by trained researchers. All methods were performed in accordance with the guidance provided in the Declaration of Helsinki.

### Study context

 Singapore is a small island country with a total population of around 5.64 million. Being a multi-ethnic nation, it is comprised of Chinese (74.3 %), Malays (13.4 %), Indians (9.0 %), and other ethnicities (3.2 %) [26]. Singapore has a mixed healthcare system with the public, private, and non-profit institutions delivering acute, primary, intermediate, and long-term care services. The role played by family members in providing care to their loved ones is in line with the Singaporean principles of families being the “*first line of support*” with the community and government stepping in where necessary [27].

### Participants

 The eligibility criteria for stroke survivors were Singaporean or permanent resident, at least 21 years and above at the time of recruitment, stroke being diagnosed by a clinician and/or supported by brain imaging and able to participate in the interview. For caregivers, we included individuals aged 21 years and above, who were either an immediate family member, extended family member or friend, were recognized as the main person offering care and taking responsibility for the patient, as recognized by the patient and not paid for caregiving. Paid or professional caregivers and those refusing to audio-record the interview were excluded from the study. Stroke survivors and family caregivers were purposively sampled across different caregiver identities (i.e., spouse, adult-child, sibling and others inclusive of distant relatives or friends). Recruitment was conducted across 3 recruitment sites, namely, outpatient rehabilitation setting, outpatient clinic setting and support organization for stroke survivors and their caregivers.

### Data Collection

 Fifty interviews were conducted involving 61 participants (35 caregivers and 26 stroke survivors) from October 2018 to February 2019, at which point thematic saturation was reached. The interview guide for both caregivers and stroke survivors is detailed in Supplementary File 1. The interviews generally lasted between 28 and 58 min, with the longest lasting 129 min, and were conducted in either English, Mandarin, Malay or Tamil. The principal investigator (ST), a public health trained physician pursuing her Ph.D. in health services research at the time of this study, had prior training and experience conducting qualitative research, including stroke survivors and caregivers as participants. ST and other researchers involved in data collection (ASLC, AF, ZBL) had no prior relationship with the participants. The research team comprised of academics, neurologists, physiotherapists, occupational therapists, research associates, and research assistants, with two members having prior experience caregiving for a family member with disability (not stroke related). Field notes were taken during the interviews and memos written after the completion of the interview. We also collected socio-demographic information on stroke survivors and caregivers. Recorded interviews were transcribed and translated to English (where applicable). We removed participant identifiers and assigned an identity code for each transcript to maintain participant confidentiality. NVivo 12 software was used for data management and the facilitation of data analysis [28].

### Data analysis

We followed Braun and Clarke’s guidance on conducting thematic analysis which comprised of the following six steps: familiarizing ourselves with our data, generating initial codes, searching for themes, reviewing of themes, defining and naming themes and producing the report [29]. To explore the differences in themes and sub-themes across different caregiver identities (i.e., spouse, adult-child), we ran multiple matrix coding queries in NVivo 12, which allowed us to explore coding intersections across two items (e.g., coded sections and categorical variable of caregiver identity) [30]. Summarized findings of matrix coding queries are provided in Table 1.

**Table 1:** Illustration of matrix coding query across different coded references and caregiver identities

Coded References (Coded References as Proportion of total sample of caregiver type)	Spouse	Adult-child	Sibling	Others
**Theme A.1. Factors influencing seeking care**				
**Subtheme A.1.1. Financial factors**	15 (1.15)	8 (0.44)	3 (1.50)	2 (1.00)
**Subtheme A.1.2. Structural or healthcare system related factors**	27 (2.08)	38 (2.11)	5 (2.50)	4 (2.00)
**Theme A.2. Decision to seek care**				
**Subtheme A.2.1. Caregiver decides**	9 (0.69)	35 (1.94)	6 (3.00)	1 (0.50)
**Subtheme A.2.2. Not one person’s decision to make**	8 (0.62)	27 (1.50)	2 (1.00)	2 (1.00)
**Theme A.3. Role of caregiver in seeking care**				
**Subtheme A.3.1. Recognizing symptoms**	17 (1.31)	6 (0.33)	0 (0)	0 (0)
**Subtheme A.3.2. Coordinating care**	0 (0)	3 (0.17)	3 (1.50)	0 (0)
**Subtheme A.3.3. Accompanying the stroke survivor for healthcare appointments**	6 (0.46)	12 (0.67)	0 (0)	3 (1.50)
**Theme B.1. Service around the patient**				
**Subtheme B.1.1. Patient choice or preference**	16 (1.23)	8 (0.44)	8 (4.00)	0 (0)
**Subtheme B.1.2. Dignity and respect**	8 (0.62)	12 (0.67)	0 (0)	0 (0)
**Theme B.2. Service with care**				
**Subtheme B.2.1. Communication during a healthcare encounter**	33 (2.54)	27 (1.50)	1 (0.50)	3 (1.5)
**Subtheme B.2.2. Trust in the healthcare system**	8 (0.62)	6 (0.33)	5 (2.50)	0 (0)
**Subtheme B.2.3. Personal touch experienced in a healthcare encounter**	17 (1.31)	9 (0.50)	0 (0)	0 (0)
**Theme B.3. Role of caregiver in healthcare encounters**				
**Subtheme B.3.1. Advocate for the stroke survivor**	15 (1.15)	5 (0.28)	14 (7.00)	1 (0.50)
**Subtheme B.3.2. Active participant in healthcare encounter**	6 (0.46)	16 (0.89)	6 (3.00)	2 (1.00)

We used the parallel criteria by Lincoln and Guba [31–33] to guide the development of study processes and report on the trustworthiness of our findings. We practised peer debriefing during data collection to discuss insights gained by different team members and during group discussions to share preliminary themes and gain consensus on findings. A proportion of transcripts were co-analysed with a peer researcher. Additionally, researchers practised reflexivity and maintained an audit trail throughout the conduct of the study. Findings are reported in accordance with the COREQ guidelines [34]. (Please refer Additional File 1).

## Results

**Fig. 1:**
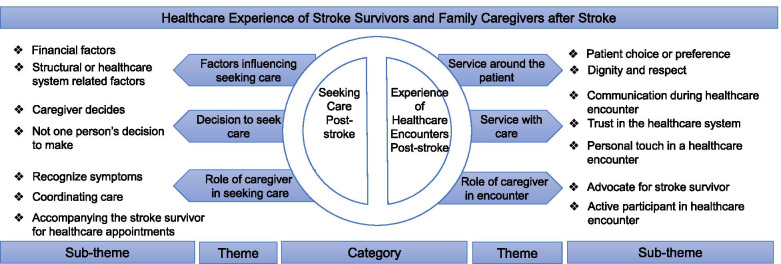
Summary Diagram of main Findings

We summarized our findings of healthcare experience post-stroke across two categories: seeking care and experience of healthcare encounters. Within the category of seeking care, following themes were coded: factors influencing seeking care with sub-themes of financial, and structural factors; decision to seek care with sub-themes of caregiver decides, and not one person’s decision to make; and role of caregiver in seeking care with sub-themes of recognize symptoms, coordinate care and accompanying to healthcare appointments. Within the category of experience of healthcare encounters, following themes were coded: service around the patient with sub-themes of patient choice or preference, and dignity and respect; service with care with sub-themes of communication during a healthcare encounter, trust in the healthcare system and personal touch experienced during a healthcare encounter; and role of caregiver in healthcare encounters with sub-themes of advocate for the stroke survivor, and active participant in the healthcare encounter (Fig. 1).

The sample comprised of 35 caregivers and 26 stroke survivors. (Please refer to Table 2) The caregivers were between 22 and 80 years old, with more than half female, married, and living with their spouses. Twenty-three were Chinese, 9 were Malay, and 3 were Indian. There were 18 adult-child caregivers, 13 spousal caregivers, 2 each of siblings and other caregivers. The stroke survivors were between 45 and 84 years old, with more than half males. Thirteen were Chinese, 9 were Malay, and 3 were Indian.

**Table 2:** Demographic characteristics of participants

**Demographic characteristics**	n
**CAREGIVER (N=35)**	
**Age (in years)**	
- **Range**	22 - 80
**Gender**	
- **Male**	13
- **Female**	22
**Ethnicity**	
- **Chinese**	23
- **Malay**	9
- **Indian**	3
**Marital status**	
- **Married living with spouse**	23
- **Single never married**	10
- **Divorced**	1
**Education**	
- **Primary**	8
- **Secondary**	6
- **Post-secondary**	10
- **University**	10
**Employment**	
- **Working part-time**	6
- **Working full-time**	14
- **Unemployed**	5
- **Homemaker**	7
- **Retired**	3
**Caregiver Identity**	
- **Spouse**	13
- **Adult-child**	18
- **Sibling**	2
- **Others**	2
**Co-residing status**	
- **Yes**	28
- **No**	6
**Caregiving duration (in months)**	
- **Mean (SD)**	46.8 (46.9)
**First experience caregiving**	
- **Yes**	29
- **No**	5
**Number of other family caregivers involved in caregiving**	
- **0**	11
- **1**	6
- **2**	8
- **3**	5
- **4**	3
- **6**	1
**STROKE SURVIVOR (N=26)**	
**Time since stroke (in years)**	
- **Mean (SD)**	3.5 (3.3)
**Index stroke recurrent**	
- **Yes**	6
- **No**	19
**Age (in years)**	
- **Range**	45 - 84
**Gender**	
- **Male**	17
- **Female**	8
**Ethnicity**	
- **Chinese**	13
- **Malay**	9
- **Indian**	3

Numbers may not add up to total because of missing data

## A. Seeking care post-stroke

### Theme A.1. Factors influencing seeking care

#### Subtheme A.1.1. Financial factors

Financial factors were almost twice as commonly reported by spousal caregivers as compared to adult-child caregivers. Participants described opting for services that were affordable or subsidized, especially since most of the stroke survivors required long term follow-up in outpatient settings for chronic diseases. Some of the participants commented on the limited affordability of rehabilitation, sharing that current subsidies may not be enough to meet the needs of sustained rehabilitation.

#### Subtheme A.1.2. Structural or healthcare system related factors

Structural factors influencing the decision to seek healthcare services were as follows: waiting time, access to services, the ambience of healthcare settings, processes of care, and resources available. Long waiting time was reported as a common barrier to accessing services, and often caregivers would choose an alternative option, where feasible. Access to services included transport related challenges that stroke survivors and their caregivers encountered while accessing health care services.

### Theme A.2. Decision to seek care

The caregiver deciding to seek care was the most commonly reported scenario closely followed by the decision made by more than one person.

#### Subtheme A.2.1. Caregiver decides

Consideration of expenses was a common occurrence when caregivers were deciding to seek care. Public primary care setting was considered an affordable alternative to private primary care setting, based on the available subsidies for medicines, which was a recurring cost as most stroke survivors were on chronic medications. In instances where the caregiver was the main decision maker, rescheduling of appointments for stroke survivors to match their other commitments was common. Most commonly, caregivers made the healthcare seeking decisions in the primary care context, with rehabilitation setting as the second most commonly reported setting, with perceived effectiveness being a consideration for such decisions. Interestingly, a small proportion of adult-child caregivers reported not being certain at times of where to seek care and at what time points, as learning to care seemed to be a continuous process.

#### Subtheme A.2.2. Not one person’s decision to make

In many instances, often, the decision to seek care was taken jointly by more than one stakeholder (i.e., caregiver, stroke survivor, healthcare provider, or other family members). Adult-child caregivers were most commonly reported as being in collaborative healthcare seeking decisions. Participants reported three types of shared decision making arrangements: caregiver with a healthcare provider, caregiver with stroke survivor, and caregiver with inputs from other family caregivers. Healthcare providers were mostly part of the decision to seek outpatient care services, whereas the stroke survivor was part of the decision to continue rehabilitation services. Other family members were involved in the decision to seek care based on the caregiving arrangement. Shared caregiving arrangements with distributed caregiving responsibilities, commonly observed in adult-child caregivers, meant they would often discuss and come to a conclusion for service seeking.

### Theme A.3. Role of caregiver in seeking care

The main sub-themes under this theme were recognizing symptoms, coordinating care, and accompanying the stroke survivor for the healthcare appointments.

#### Subtheme A.3.1. Recognizing symptoms

Spousal caregivers described recognizing symptoms more commonly as compared to adult-child caregivers. Most of the shared accounts detailed the stroke survivor’s symptoms related to the index stroke event, such as, “*limping on the left side*”, “*he is different*”, “*cannot stand and she cannot move her hand*” and so forth.

#### Subtheme A.3.2. Coordinating care

Adult-child caregivers were more commonly involved in the role of coordinating care for their stroke survivors. Efforts to coordinate care across different care settings were reported as challenging by some, with little room for healthcare professionals to accommodate. While the caregivers reported coordinating on their end and taking turns to go to these multiple appointments, it was stressful and took a toll on a few.

#### Subtheme A.3.3. Accompanying the stroke survivor for healthcare appointments

Along with the subtheme of coordinating care, this theme of accompanying the stroke survivors for healthcare appointments was more commonly reported by adult-child caregivers as compared to spousal caregivers.

## B. Experience of healthcare encounters post-stroke

### Theme B.1. Service around the patient

#### Subtheme B.1.1. Patient choice or preference

Both patients and caregivers opined that choice of healthcare professional is something they do not have in the public healthcare setting, both acute and outpatient care, as expressed by a stroke survivor, “*Only the Polyclinic is a bit like buying a lottery ticket*” (P10, 62 years, male, stroke survivor).

Choice had another dimension within the acute care setting, which was autonomy; the participants recounted their experiences in an inpatient setting, highlighting they felt powerless in the way they were treated. They preferred to have a say in what happened to them. Participants generally described their experience in an outpatient rehabilitation setting in a more positive light, where they shared accounts of stroke survivors being central to management and therapy decisions.

#### Subtheme B.1.2. Dignity and respect

 The sub-theme of dignity and respect included instances where the participants mentioned their expectations of being treated with courtesy and maintaining their dignity. Participants had certain expectations such as “*must see the body language*”, “ *read it and do your homework before coming*”, “*talk to people clearly*” and so forth. Another dimension of this sub-theme was the lack of privacy in inpatient settings. As a stroke survivor, the individual has some physical limitations which necessitate assistance from others in ADL such as showering, hygiene, toileting, and feeding. Some of the stroke survivors felt that their privacy was not respected during such encounters.

### Theme B.2. Service with care

Service with care theme captures the essence of softer relational dynamics that stroke survivors and caregivers experienced across different healthcare settings.

#### Subtheme B.2.1. Communication during a healthcare encounter

 This included understanding the content of the conversation with the healthcare provider, and also how the participants were spoken to. Stroke survivors and caregivers often came to these healthcare encounters with certain expectations, and when these were not met, they resulted in a negative perspective of the healthcare encounter. While sharing of information was a common thread in accounts of spousal and adult-child caregivers, spousal caregivers also valued the manner in which they were spoken to. Secondly, while adult-child caregivers had no problem understanding the content of such conversations, few of the spousal caregivers struggled.

#### Subtheme B.2.2. Trust in the healthcare system

Based on past healthcare experiences, participants had their guard up mostly for inpatient setting, during their more current healthcare encounters with concerns around trusting the healthcare professionals or care received. A sibling caregiver recounted her past experiences in inpatient setting, which seemed to shape her current perspective of inpatient services. She emphasized the vulnerable state of a stroke survivor in inpatient setting, and the need to be vigilant ensuring their safety. Reported lack of trust on healthcare professionals, made a stroke survivor talk about the need to educate oneself to manage care received and how ignorance may not be the best approach to address care in an inpatient setting.

We found sub-theme of communication and trust being interrelated. Communications involving limited symptom clarity and explanation from healthcare professionals seemed to result in participants having limited trust in the advice given to them. Not understanding the rationale for some diagnostic tests (which cost money) seemed to make a spousal caregiver and stroke survivor question the intention behind such healthcare decisions in an outpatient setting.

#### Subtheme B.2.3. Personal touch experienced in a healthcare encounter

The described instances of personal touch or valuing care beyond medical management, mostly occurred in a step-down inpatient setting as compared to the outpatient setting. Participants recounted instances of friendly reassurance by the healthcare professionals, participating in their celebrations like birthdays, consoling them in their unfortunate moments, helping out with financial problems (e.g., arranging for a wheelchair) and so forth.

### Theme B.3. Role of caregiver in healthcare encounters

#### Subtheme B.3.1. Advocate for the stroke survivor

Though advocacy could manifest in a myriad of ways, for the current analysis, to be an advocate meant always keeping the interest and well-being of their care recipient to the forefront during each healthcare encounter. This ranged from trying to make the processes of care more person-centered by giving feedback, to asking for delayed discharge from inpatient tertiary setting to prepare for the new caregiving role, to consulting the physician on clinical management of their loved ones to fighting for what the caregiver felt was best for the stroke survivor. Advocacy was mostly prominent in the rehabilitation setting, as the caregivers tried to get the best rehabilitation services for the stroke survivor.

#### Subtheme B.3.2. Active participant in healthcare encounter

This sub-theme included instances where participants reported caregiver being involved in giving and receiving information from the healthcare provider on the stroke survivor’s behalf and participating in the consults by contributing to the management discussions, where possible. Adult-child caregivers were more active in giving and receiving information and in participating in the consults as compared to spousal caregivers. Caregivers wanted healthcare professionals to talk to them about stroke survivors’ health status, changes in health status, decisions around the change of care settings, and so forth. Some caregivers also reported wanting to be considered as a partner in the post-stroke recovery journey, for instance, provision of counselling for them to cope.

## Discussion

We described the healthcare experiences of family caregivers and stroke survivors post-stroke across two categories of seeking care and experience of healthcare encounters. Multi-dimensional role of caregivers in healthcare experience emerged as one of the major findings, along with differences in themes between spouse and adult-child caregivers contributing new knowledge. (Refer Table 3 for summary of themes, illustrative quotes and differences across caregiver identities)

**Table 3:** Summary of themes with illustrative quotes & differences across different caregiver identities

[A] Seeking healthcare services post-stroke			
**Themes**	**Sub-themes**	**Illustrative quotes**	**Differences across caregiver identities**
**A.1. Factoring influencing seeking care**	A.1.1. Financial	*All these hospital appointments, procedures, and you know medicine, you can only use a certain amount. In-patient, you can claim Medisave, you can claim insurance. But outpatient? He has used up his, he has maxed out his outpatient claim for him. So now, although he is subsidized, but for every clinic appointment, every procedure done, we still have to pay. Like last month alone uh, his out-patient bill was what, about a thousand over dollars. (C10, 49 years, female, spouse caregiver)*	- Financial factors were almost twice more commonly reported by spouse caregivers as compared to adult-child caregivers
	A.1.2. Structural or healthcare system related	*Polyclinics I don’t know. The reason is it’s long queue so like I’ll bring her down because she saw the Family Clinic, let’s say for fever or whatever, so even like before it happened, I seldom go to Polyclinic because of the long queue. I just cannot afford to. I cannot spare three hours there queueing. Just go downstairs in the Family Clinic. That’s it*. (C29, 54 years, male, adult-child caregiver)	- Waiting times and ambience of healthcare services more commonly reported by adult-child caregivers who were generally working - Access to services more commonly reported by spouse caregivers who were generally older than adult-child caregivers
**A.2. Decision to seek care**	A.2.1. Caregiver decides	*We also don’t want to put her somewhere far because it would be very hectic to travel. ABC (name of Nursing Home) near my house was full. We left very few choices. (C19, 43 years, female, adult-child caregiver)*	- Most commonly reported decision-making scenario discussed was the caregiver deciding to seek care
	A.2.2. Not one person’s decision to make	*I think it’s not my decision, let’s put it this way. I think it has to be also between my sis and myself…* (C16, 53 years, female, adult-child caregiver)	- Adult-child caregivers more commonly reported decision-making scenario where seeking healthcare was based on inputs from multiple stakeholders
**A.3. Role of caregiver in seeking care**	A.3.1. Recognize symptoms	*And then he call me then I see him different lah. His mouth is so different, I was in there. I called the ambulance*. (C04, 53 years, female, spousal caregiver)	- Spousal caregivers described recognizing symptoms more commonly than adult-child caregivers
	A.3.2. Coordinate care	*It is very good. It’s very fast. In fact, I asked the XX Hospital doctor to transport my mom all the way in YY (step-down care facility) and they said no. They need my mother to go to XX Hospital (- -) Yes. But to send my mother to XX and YY, we take turns.* (C03, 58 years, male, adult-child caregiver)	- Adult-child caregivers were more involved in the role of coordinating care as compared to spousal caregivers
	A.3.3. Accompany to appointments	Normally I cannot handle my mother alone, normally my sis will come with me, but if she is not available, then I ask my brother (- -) to transfer her. (C19, 43 years, female, adult-child caregiver)	- Adult-child caregivers more commonly highlighted accompanying the stroke survivor for healthcare appointments as compared to spousal caregivers
**[B] Experience of healthcare encounters post-stroke**			
**Themes**	**Sub-themes**	**Illustrative quotes**	**Differences by caregiver identities/types**
**B.1. Service around the patient**	B.1.1. Patient choice or preference	*We cannot say anything lah. That’s why I go. I go also. Sometimes I ruin their mood. I say, I don’t want to go ladies shower, what to do? When I talk, they’ll talk. It’s all education na*. (P01, 76 years, male, stroke survivor)	- More commonly reported by spousal caregivers as compared to adult-child caregivers
	B.1.2. Dignity and respect	Stroke Patient: *They don’t know. I’m like a, like a guinea pig. Every morning coming to like, asking me things. “Yeah, I’ll be there.” “How’s everything up here?” There’s more people coming*. (P10, 62 years, stroke survivor) Caregiver: *Yes. It’s quite basic and all that lah but, but like I say, give him a choice. They didn’t give him.* (C10, 49 years, female, spouse caregiver)	- Similar across both spouse and adult-child caregivers
**B.2. Service with care**	B.2.1. Communication during a healthcare encounter	*So my pastor stay but very difficult. Sometimes the doctor say, I cannot understand because I am not very educated you know. And the doctor say, and I can’t understand.* (C01, 70 years, female, spousal caregiver)	- Importance of sharing information was a common thread across both spouse and adult-child caregivers - Spouse caregivers valued more the manner in which they were spoken to as compared to adult-child caregivers - While adult-child caregivers had no problem understanding the content of communications with healthcare professionals, spouse caregivers struggled in some instances
	B.2.2. Trust in the healthcare system	*I said, “you know the reason of this?” Ignorance is not bliss and the doctor will kill you. (Laughs 5:03) or the medicine, that can kill you. (P09, 55 years, female, stroke survivor)*	- Similar across both spouse and adult-child caregivers
	B.2.3. Personal touch experienced in a healthcare encounter	*They say, “You also must come.” I said, “Okay lah.” I went inside there, I saw the, the decoration, the birthday. I know. So wonderful. All the staff make so happy my husband. So they give teddy bear present. Then they write “We love you all!” So my husband is so happy.* (C01, 70 years, female, spousal caregiver)	- Personal touch was more commonly reported by spousal caregivers as compared to adult-child caregivers
**B.3. Role of caregiver in healthcare encounters**	B.3.1. Advocate for the stroke survivor	*I say, “What can you offer there?” It’s totally different from the one at hospital or at the step-down care facility. They don’t want to give me the hospital, so yeah. “You don’t want to give me hospital, I’ll take the step-down care facility. But I’m not willing to go for the home type of exercises.”* (C04, 53 years, female, spousal caregiver)	- Spousal caregivers more commonly described their roles as advocates for the stroke survivors as compared to other caregivers
	B.3.2. Active participant in healthcare encounter	*I was briefing the doctor everything: what she took before, when she choked, blah, blah and everything. They say we have to put her on observation, and we will call you when the ward is ready. I said, okay that is fine. (C27, 51 years, female adult-child caregiver)*	- Adult-child caregivers were more active in giving and receiving information - Importance of sharing with the caregivers was most commonly highlighted by adult-child caregivers

Our findings demonstrate the diverse roles caregivers play in the healthcare journey of stroke survivors, including symptom recognition, coordination of care, accompanying for healthcare encounters, advocacy and active participation in healthcare encounters. Researchers have described the caregiver’s role as a qualitatively constructed social process of “*seeking*,” which they have described as *“carers’ efforts to understand their role better and to begin to establish some degree of ‘balance’ within their new and often confusing situation*” [35]. Seeking can take the form of advocacy, information exchange, participation in consult and quality assurance of healthcare services [35]. In concordance with our findings, Shafer and colleagues [36] reported caregivers to assume different roles during the healthcare journey of stroke survivors, e.g., advocates, motivators, therapists, and guardians. The authors further elaborated that caregivers assume different roles to fill in the perceived gap in healthcare services to optimize the well-being of stroke survivors. Spousal caregivers in our study reported instances where they adopted the advocacy role to get the best care for their loved ones. Similar advocacy efforts are reported by other researchers with caregivers in this study describing how “*they negotiated and had to push to receive proper help and make things happen*” [37]. This led to a sense of not being involved in the care of the stroke survivor and feeling frustrated with the care process [37]. Having established that family caregivers have multiple roles in the healthcare experience of stroke survivors based on shared accounts by the caregivers, it is important to acknowledge their contribution as being unrecognized for their effort can lead to dissatisfaction [38]. Moreover, fulfilling such caregiving tasks in the healthcare setting may exacerbate the caregiver’s perceived burden [39]. Along the same lines, one of the commonly reported caregiver needs post-stroke in the healthcare setting is for the professionals to provide education, rehabilitation, treatment and care with respect for the stroke patient. Another commonly reported caregiver need was to have information on the management and recovery trajectory of the stroke survivors [40]. Understanding such needs of the caregivers and providing adequate support in the form of information, education, training or family-centred care provision will help caregivers manage their caregiving role within and beyond the healthcare setting successfully and not be burdened. This in turn will contribute towards a better quality of life for the caregivers since caregiver burden is an established determinant of poor quality of life of caregivers [41, 42].

Another interesting finding as per the participants’ accounts was the perceived central role of family caregivers in the healthcare decision-making for stroke survivors, sometimes being reported as the primary decision makers, and on other occasions deciding with inputs from other stakeholders. This is in contrast to the common expectation in the Western setting, where the patient is expected to be the main decision maker in healthcare settings. A possible explanation for this difference may be rooted in our Eastern setting with predominant Asian values of reliance on one another and one generation supporting the other, within which family members are expected to take care of their loved ones. To this effect, a qualitative study involving older stroke survivors in China explored the decision-making process post-stroke and reported the phenomenon of “*hiding*” based on the Chinese cultural principle of “*keeping the peace*” [43]. The authors suggested that family members tend to maintain peaceful ambience with solely shouldering the responsibility of healthcare related information exchange and decision making to reduce the burden of their care recipients. This may be the case in our setting, with caregivers seemed to play a predominant decision-making role in many instances [43]. A recent conceptual analysis exploring decision-making in the healthcare setting reported decision-making to be dependent on the person fulfilling some complimentary roles e.g., advocacy, being the “*hub of information*”, possessing emotional resilience etc [44]. To contextualize to our findings, meeting all these criteria could be a rather complex requirement, resulting in scenarios where more than one person is engaged in the healthcare decision-making or as we reported that “*it is not one person’s decision to make*”.

While authors have previously reported differences across spousal and adult-child caregivers’ caregiving experience [24, 45], such differences in healthcare experience of caregivers are unexplored. To the best of our knowledge, we are among the first few to establish the diverse role of family caregivers in the healthcare experience of their stroke survivors, highlighting the differences across different types of caregivers. In our study, spousal caregivers seemed to prefer a relational healthcare experience characterised by softer aspects of communication and personal touch. For adult-child caregivers, the healthcare encounters were more transactional characterised by coordinating appointments, an information exchange with healthcare professionals, and active participation in such encounters. While financial and access factors were more salient for the spousal caregivers, waiting times and ambience of healthcare settings were more salient for adult-child caregivers. Financial factors being more commonly reported by spousal caregivers as compared to adult-child caregivers is concordant with existing literature on spousal caregivers more commonly reporting financial barriers or burden as compared to adult-child caregivers [45]. Though caregivers were commonly reported to be in a decision-making role, adult-child caregivers seemed to engage in collaborative decision-making with inputs from others involved in their caregiving arrangement. While not all the differences across caregiver identities can be discussed in relation to the age of the caregivers, we can draw some parallels across selected findings. While spousal caregivers in our study shared that they more commonly valued the manner of communication during the healthcare encounter, this was not the case with adult-child caregivers who reported no problems understanding the information shared by the healthcare providers. One possible explanation could be related to the age of the caregivers with spousal caregivers being older than adult-child caregivers. A qualitative study in Canada reported age-related differences in rehabilitation experiences of caregivers of stroke survivors, with younger caregivers highlighting the relevance of informational and training support while older caregivers did not [46]. This is also aligned with our finding of adult-child caregivers reporting to be more actively involved in healthcare encounters, giving and receiving information. Although not reported in the caregiver population, a qualitative study in Norway involving older patients reported older patients in inpatient setting experiencing poor communication, lack of clear information and coordination [47]. Another study reported that patients’ perception of communication with healthcare providers varied with age of the patients [48]. In fact, younger patients were reported to value expediency, efficiency and autonomy in their healthcare encounters, while older patients valued continuity of care and relational aspects in their healthcare encounters [49]. It is likely that age-related differences in the perception of healthcare encounters observed in patients may also be present to some extent in the healthcare encounters experienced by caregivers of stroke survivors in our study.

Participants in our study reported different experiences across different healthcare settings, with rehabilitation experience being relatively more positive as compared to inpatient experience. Along the same lines, there is a consensus in the literature on stroke survivors being satisfied with rehabilitation services across different settings [36, 50–53]. Similarities can be drawn between our findings of inpatient setting and a UK based study, with the latter providing healthcare providers’ perspectives on working in a high pressure, efficiency driven setting with prioritization of performance indicators, resulting in limited emphasis on holistic management of stroke survivors. Also, transient nature of inpatient stay meant limited opportunities for interpersonal communication and relationship building [54]. Similarly, participants in a Hongkong based study reported “*care gaps*” during hospitalization, with unmet expectation of emotional support beyond “*physical care*” [55].

The healthcare encounters not only comprised of caregivers playing a central role in multiple capacities as per the participants’ accounts, but also themes of “*service around the patient*” and “*service with care*”, highlighting the importance of having stroke survivor and family caregiver dyads at the centre of any healthcare experience post-stroke [19], with healthcare systems adopting a family-centred approach across all care settings [56]. Patient- and Family-centred care is defined as “*an approach to the planning, delivery, and evaluation of healthcare that is grounded in mutually beneficial partnerships among healthcare providers, patients, and families*” [57]. We reported participants valued softer aspects in healthcare encounters under the theme of “*service with care*”, and also spousal caregivers seem to express such views more frequently than adult-child caregivers. A qualitative study in Norway reported similar findings ranging from being treated with humanity to respecting patient autonomy, communication during patient-professional encounters and trust in professionals [51]. While this study was limited to views of stroke survivors in a rehabilitation setting, we provided views of both stroke survivors and family caregivers across different healthcare settings. Interestingly, preference for such softer aspects of healthcare encounters seems to transcend geographical boundaries, whereas having a caregiver in healthcare decision-making role may present as a more heterogenous concept with differences across Asian and Western settings. In concordance with our finding of perception of limited communication being associated with limited trust in the healthcare provider, White and colleagues [58] also reported insufficient communication altering stroke survivors’ confidence in the healthcare processes. We found past experiences shaped participants’ trust in acute inpatient healthcare setting, which is in alignment with similar findings reported elsewhere [59]. Policy recommendations from our findings are outlined in Table 4.

**Table 4:** Policy recommendations

Theme	Sub-theme	Policy recommendations
**[A] Seeking healthcare services post-stroke**		
**A.1. Factoring influencing seeking care**	A.1.1. Financial	Provision of financial counselling to caregivers, especially spousal caregivers, providing information on subsidies available
	A.1.2. Structural or healthcare system related	Provision of affordable transport related information/support to family caregivers, especially spousal caregivers
**A.2. Decision to seek care**	A.2.1. Caregiver decides	Engage caregivers to optimise the use of primary care and outpatient rehabilitation services; making caregivers partners in stroke survivor’s healthcare journey to keep them motivated to continue seeking services for stroke survivors Inform caregivers about stroke survivors’ care needs and healthcare appointments; provide a direct line with a care coordinator or designated personnel who can be contacted at times when caregivers are not sure of the healthcare setting they should visit during “crises”
	A.2.2. Not one person’s decision to make	Healthcare providers should inquire about and be aware of caregiving arrangement of stroke survivors and engage the main caregiver in healthcare information exchange and decision making or ensuring the information is passed on to the relevant person(s) Training healthcare professionals in successfully holding family conferences, where needs and ethical dimension of including family members are factored into healthcare decisions of stroke survivors
**A.3. Role of caregiver in seeking care**	A.3.1. Recognize symptoms	Educate the caregivers, especially spousal caregivers, about symptoms or red flags to look for in stroke survivors in community setting
	A.3.2. Coordinate care	Inform and educate caregivers, especially adult-child caregivers, on healthcare services available and subsequent scheduled appointments during each healthcare encounter
	A.3.3. Accompany to appointments	Scheduling appointments for stroke survivors taking into consideration the availability of family caregivers (and their other commitments like work), especially adult-child caregivers, to reduce rescheduling or no show at booked appointments
**[B] Experience of healthcare encounters post-stroke**		
**B.1. Service around the patient**	B.1.1. Patient choice or preference	Ensure principles of person-centred care are embedded in healthcare system, especially in acute care setting, with due consideration to patient choice
	B.1.2. Dignity and respect	Adoption of holistic management approach in both training and practice, which is inclusive of biopsychosocial elements, seeing stroke survivors as individuals, enabling them to maintain their dignity and giving them space during vulnerable moments
**B.2. Service with care**	B.2.1. Communication during a healthcare encounter	Healthcare professionals should invest time in communication aspects of a consult with sharing of information with the stroke survivors and their caregivers in a simplified manner, ensuring they understand Tailoring of communication in a healthcare encounter to the needs of different types of caregivers, keeping in mind that spousal caregivers value relational aspects (e.g., the manner they are spoken to and so forth) and adult-child caregivers value transactional aspects (e.g., timely sharing of information and technical content and so forth)
	B.2.2. Trust in the healthcare system	Ensuring stroke survivors and family caregivers have a good healthcare experience aligned with their expectations, so that the trust in the healthcare system is maintained, especially in acute care setting Strategies to improve communication between healthcare professional and stroke survivor and family caregivers will facilitate building and maintaining of trust
	B.2.3. Personal touch experienced in a healthcare encounter	Inclusion of empathy and compassion training in medical curriculum and promoting healthcare professionals to practice learnt principles in healthcare encounters with stroke survivors and their family caregivers
**B.3. Role of caregiver in healthcare encounters**	B.3.1. Advocate for the stroke survivor	Incorporate feedback given by family caregivers towards improving processes of care and making healthcare delivery more “patient- and family-centred”
	B.3.2. Active participant in healthcare encounter	Promote partnership across healthcare professionals, stroke survivors and family caregivers towards collaborative care for the stroke survivors

### Strengths

Our study has several strengths. We are among the first to the best of our knowledge to describe the multi-dimensional role of family caregivers in healthcare experience of stroke survivors along with highlighting the differences across spouse and adult-child caregivers. Our findings have provided context to the previously reported quantitative finding of caregiver identity being associated with hospitalizations after a stroke [19]. Our sample was diverse, including both stroke survivors and different types of family caregivers from three different settings, making our results more comprehensive and inclusive. Language was not a barrier and we managed to conduct interviews with English, Mandarin, Malay, and Tamil speaking participants. Our study was conducted and reported following the trustworthiness criteria recommended for qualitative studies [32, 33].

### Limitations

 Following are some of the limitations of our study. It was challenging to recruit siblings and other (i.e., distant relatives and friends) types of caregivers as we wanted to compare the themes across all four types of caregivers. However, our results are limited to spousal and adult-child caregivers due to limited recruitment of sibling (N=2) and other (N=2) caregivers. Our findings would potentially be transferrable across spousal and adult-child caregivers only. We did not perform member checking with our participants as we did not retain contactable information beyond the interview stage. Moreover, we did not want to inconvenience our participants considering our sample did not include healthy individuals.

## Conclusions

 With the aim to describe the healthcare experiences of family caregivers and stroke survivors across different caregiver identities, we highlighted the multi-dimensional role of caregivers in the post-stroke healthcare experience. Unique to Asian context, as per the participants’ accounts, family caregivers seemed to play a central role in healthcare seeking decisions for stroke survivors. For adult-child caregivers, collaborative decision-making with inputs from multiple stakeholders was commonly reported. While spousal caregivers preferred the relational aspects of healthcare encounters, for adult-child caregivers, healthcare encounters were mainly transactional involving their active participation. Differences in healthcare experiences across different caregiver identities should be incorporated into tailoring a healthcare experience suited for the type of end-users. Other practical implications would be acknowledging caregivers as partners in post-stroke healthcare journey, provision of training and supportive decision-making environment for caregivers, reinforcing communication aspects in the medical, nursing and allied healthcare curriculum to improve experience of healthcare encounters and build trust between the healthcare professional and stroke survivor-caregiver dyads.

## Supplementary Information

**Additional file 1.**

**Additional file 2.**

## Data Availability

The datasets used and analysed during the current study are available from the corresponding author on reasonable request.

## Abbreviations

ADL: activities of daily living
IADL: instrumental activities of daily living

## References

[1] Miller EL, Murray L, Richards L, Zorowitz RD, Bakas T, Clark P (2010). Comprehensive overview of nursing and interdisciplinary rehabilitation care of the stroke patient: a scientific statement from the American Heart Association. Stroke.

[2] Hartman-Maeir A, Soroker N, Ring H, Avni N, Katz N (2007). Activities, participation and satisfaction one-year post stroke. Disability and rehabilitation.

[3] Thom T, Haase N, Rosamond W, Howard VJ, Rumsfeld J, Manolio T (2006). Heart disease and stroke statistics--2006 update: a report from the American Heart Association Statistics Committee and Stroke Statistics Subcommittee. Circulation.

[4] Sterling MR, Shaw AL. Sharing the Care-A Patient and Her Caregivers. JAMA Internal Med. 2019;179(12):1617. 10.1001/jamainternmed.2019.4231 31566655

[5] Caregiver Statistics: Demographics.: Family Caregiver Alliance 2016 [Available from: https://www.caregiver.org/caregiver-statistics-demographics.

[6] Lacey RE, McMunn A, Webb E (2019). Informal caregiving patterns and trajectories of psychological distress in the UK Household Longitudinal Study. Psychological medicine.

[7] Skolarus LE, Freedman VA, Feng C, Wing JJ, Burke JF (2016). Care Received by Elderly US Stroke Survivors May Be Underestimated. Stroke.

[8] Joo H, Dunet DO, Fang J, Wang G (2014). Cost of informal caregiving associated with stroke among the elderly in the United States. Neurology.

[9] Bell JF, Whitney RL, Young HM (2019). Family Caregiving in Serious Illness in the United States: Recommendations to Support an Invisible Workforce. Journal of the American Geriatrics Society.

[10] Gaugler JE (2010). The longitudinal ramifications of stroke caregiving: a systematic review. Rehabilitation psychology.

[11] Greenwood N, Mackenzie A, Cloud GC, Wilson N (2009). Informal primary carers of stroke survivors living at home–challenges, satisfactions and coping: a systematic review of qualitative studies. Disability and rehabilitation.

[12] Lou S, Carstensen K, Jørgensen CR, Nielsen CP (2017). Stroke patients’ and informal carers’ experiences with life after stroke: an overview of qualitative systematic reviews. Disability and rehabilitation.

[13] Mackenzie A, Greenwood N (2012). Positive experiences of caregiving in stroke: a systematic review. Disability and rehabilitation.

[14] Sperber NR, Boucher NA, Delgado R, Shepherd-Banigan ME, McKenna K, Moore M (2019). Including Family Caregivers In Seriously Ill Veterans’ Care: A Mixed-Methods Study. Health affairs (Project Hope).

[15] Luker J, Murray C, Lynch E, Bernhardsson S, Shannon M, Bernhardt J (2017). Carers’ Experiences, Needs, and Preferences During Inpatient Stroke Rehabilitation: A Systematic Review of Qualitative Studies. Archives of physical medicine and rehabilitation.

[16] Tyagi S, Koh GC, Nan L, Tan KB, Hoenig H, Matchar DB (2018). Healthcare utilization and cost trajectories post-stroke: role of caregiver and stroke factors. BMC health services research.

[17] Roth DL, Sheehan OC, Huang J, Rhodes JD, Judd SE, Kilgore M (2016). Medicare claims indicators of healthcare utilization differences after hospitalization for ischemic stroke: Race, gender, and caregiving effects. International journal of stroke: official journal of the International Stroke Society.

[18] Ottenbacher K, Graham J, Ottenbacher A, Lee J, Al Snih S, Karmarkar A (2012). Hospital readmission in persons with stroke following postacute inpatient rehabilitation. Journals of Gerontology Series A: Biomedical Sciences and Medical Sciences.

[19] Tyagi S, Koh GCH, Luo N, Tan KB, Hoenig H, Matchar DB (2019). Dyadic approach to post-stroke hospitalizations: role of caregiver and patient characteristics. BMC neurology.

[20] White CL, Brady TL, Saucedo LL, Motz D, Sharp J, Birnbaum LA (2015). Towards a better understanding of readmissions after stroke: partnering with stroke survivors and caregivers. Journal of clinical nursing.

[21] Pindus DM, Mullis R, Lim L, Wellwood I, Rundell AV, Aziz NAA, et al. Stroke survivors’ and informal caregivers’ experiences of primary care and community healthcare services–A systematic review and meta-ethnography. PloS one. 2018;13(2):e0192533. 10.1371/journal.pone.0192533 PMC5821463 29466383

[22] Fischer R, Roy DE, Niven E (2014). Different folks, different strokes: Becoming and being a stroke family. Kai Tiaki Nursing Research.

[23] Hsu HC, Shyu YI (2003). Implicit exchanges in family caregiving for frail elders in Taiwan. Qualitative health research.

[24] Lee Y, Smith L (2012). Qualitative research on Korean American dementia caregivers’ perception of caregiving: Heterogeneity between spouse caregivers and child caregivers. Journal of Human Behavior in the Social Environment.

[25] Sandelowski M. Whatever happened to qualitative description? Res Nurs Health. 2000;23(4):334-40. 10.1002/1098-240x(200008)23:4<334::aid-nur9>3.0.co;2-g 10940958

[26] Population and Vital Statistics: Ministry of Health (MOH), Singapore; [Available from: https://www.moh.gov.sg/resources-statistics/singapore-health-facts/population-and-vital-statistics.

[27] Improving the lives of low-income and vulnerable families in Singapore Singapore: Ministry of social and family development, Singapore; 2018 [Available from: https://www.gov.sg/~/sgpcmedia/media_releases/msf/press_release/P-20181101-1/attachment/Occasional%20Paper%20-%20Improving%20the%20lives%20of%20low-income%20and%20vulnerable%20families%20in%20Singapore.pdf.

[28] NVivo qualitative data analysis software; QSR International Pty Ltd. Version 12, 2018.

[29] Braun V, Clarke V (2006). Using thematic analysis in psychology. Qualitative research in psychology. Qualitative Research in Psychology.

[30] Matrix coding query: NVIVO 12 (Windows); 2018 [Available from: https://help-nv.qsrinternational.com/12/win/v12.1.90-d3ea61/Content/queries/matrix-coding-query.htm.

[31] Guba EG. Criteria for assessing the trustworthiness of naturalistic inquiries. Ectj. 1981;29(2):75.

[32] Lincoln YS, Lynham SA, Guba EG (2011). Paradigmatic controversies, contradictions, and emerging confluences, revisited. The Sage handbook of qualitative research.

[33] Morrow SL (2005). Quality and trustworthiness in qualitative research in counseling psychology. Journal of counseling psychology.

[34] Tong A, Sainsbury P, Craig J (2007). Consolidated criteria for reporting qualitative research (COREQ): a 32-item checklist for interviews and focus groups. International journal for quality in health care.

[35] Brereton L, Nolan M. ‘Seeking’: a key activity for new family carers of stroke survivors. Journal of clinical nursing. 2002;11(1):22–31. 10.1046/j.1365-2702.2002.00564.x 11845752

[36] Shafer JS, Shafer PR, Haley KL (2019). Caregivers navigating rehabilitative care for people with aphasia after stroke: a multi-lens perspective. International journal of language & communication disorders.

[37] Backstrom B, Sundin K (2010). The experience of being a middle-aged close relative of a person who has suffered a stroke--six months after discharge from a rehabilitation clinic. Scandinavian journal of caring sciences.

[38] Kuluski K, Peckham A, Gill A, Arneja J, Morton-Chang F, Parsons J, et al. “You’ve got to look after yourself, to be able to look after them” a qualitative study of the unmet needs of caregivers of community based primary health care patients. BMC geriatrics. 2018;18(1):275. 10.1186/s12877-018-0962-5 PMC6233534 30419819

[39] Funk LM, Dansereau L, Novek S (2019). Carers as System Navigators: Exploring Sources, Processes and Outcomes of Structural Burden. The Gerontologist.

[40] Farahani MA, Bahloli S, JamshidiOrak R, Ghaffari F (2020). Investigating the needs of family caregivers of older stroke patients: a longitudinal study in Iran. BMC geriatrics.

[41] Pucciarelli G, Vellone E, Savini S, Simeone S, Ausili D, Alvaro R (2017). Roles of changing physical function and caregiver burden on quality of life in stroke: A longitudinal dyadic analysis. Stroke.

[42] Tsai Y-H, Lou M-F, Feng T-H, Chu T-L, Chen Y-J, Liu H-E (2018). Mediating effects of burden on quality of life for caregivers of first-time stroke patients discharged from the hospital within one year. BMC neurology.

[43] Wang Y, Nolan M (2016). Older people and decision-making following acute stroke in China:‘hiding’as a barrier to active involvement. Ageing & Society.

[44] Gray TF, Nolan MT, Clayman ML, Wenzel JA (2019). The decision partner in healthcare decision-making: A concept analysis. International journal of nursing studies.

[45] Pinquart M, Sorensen S (2011). Spouses, adult children, and children-in-law as caregivers of older adults: a meta-analytic comparison. Psychology and aging.

[46] Smith SD, Gignac MA, Richardson D, Cameron JI (2008). Differences in the experiences and support needs of family caregivers to stroke survivors: does age matter?. Topics in stroke rehabilitation.

[47] Lilleheie I, Debesay J, Bye A, Bergland A (2020). A qualitative study of old patients’ experiences of the quality of the health services in hospital and 30 days after hospitalization. BMC health services research.

[48] DeVoe JE, Wallace LS, Fryer GE (2009). Patient age influences perceptions about health care communication. Family medicine.

[49] Jung HP, Baerveldt C, Olesen F, Grol R, Wensing M (2003). Patient characteristics as predictors of primary health care preferences: a systematic literature analysis. Health expectations.

[50] Lamontagne ME, Richards C, Azzaria L, Rosa-Goulet M, Clement L, Pelletier F (2019). Perspective of patients and caregivers about stroke rehabilitation: the Quebec experience. Topics in stroke rehabilitation.

[51] Mangset M, Tor Erling D, Forde R, Wyller TB. ‘We’re just sick people, nothing else’: … factors contributing to elderly stroke patients’ satisfaction with rehabilitation. Clinical rehabilitation. 2008;22(9):825–35. 10.1177/0269215508091872 18728136

[52] Pindus DM, Mullis R, Lim L, Wellwood I, Rundell AV, Abd Aziz NA (2018). Stroke survivors’ and informal caregivers’ experiences of primary care and community healthcare services - A systematic review and meta-ethnography. PloS one.

[53] Salisbury L, Wilkie K, Bulley C, Shiels J. ‘After the stroke’: patients’ and carers’ experiences of healthcare after stroke in Scotland. Health & social care in the community. 2010;18(4):424–32. 10.1111/j.1365-2524.2010.00917.x 20491968

[54] Ryan T, Harrison M, Gardiner C, Jones A (2017). Challenges in building interpersonal care in organized hospital stroke units: The perspectives of stroke survivors, family caregivers and the multidisciplinary team. Journal of advanced nursing.

[55] Yeung SM, Wong FK, Mok E (2011). Holistic concerns of Chinese stroke survivors during hospitalization and in transition to home. Journal of advanced nursing.

[56] Creasy KR, Lutz BJ, Young ME, Stacciarini JM (2015). Clinical Implications of Family-Centered Care in Stroke Rehabilitation. Rehabilitation nursing: the official journal of the Association of Rehabilitation Nurses.

[57] Johnson B, Abraham M (2012). Partnering with patients, residents, and families: A resource for leaders of hospitals, ambulatory care settings, and long-term care communities.

[58] White JH, Magin P, Pollack MR (2009). Stroke patients’ experience with the Australian health system: a qualitative study. Canadian journal of occupational therapy Revue canadienne d’ergotherapie.

[59] Artuso S, Cargo M, Brown A, Daniel M (2013). Factors influencing health care utilisation among Aboriginal cardiac patients in central Australia: a qualitative study. BMC health services research.

