# Supplementary material for: Seeking healthcare services post-stroke: a qualitative descriptive study exploring family caregiver and stroke survivor perspectives in an asian setting
Source: BMC Neurol. 2021 Nov 5;21:429. doi: 10.1186/s12883-021-02463-7 (PMC8569985; doi:10.1186/s12883-021-02463-7)
Supplement: Supplementary file 1 — Additional file 1. [file 12883_2021_2463_MOESM1_ESM.pdf]

Consolidated criteria for reporting qualitative studies (COREQ): 32-item checklist

Developed from:
Tong A, Sainsbury P, Craig J. Consolidated criteria for reporting qualitative research (COREQ): a 32-item checklist for interviews and focus groups. *International Journal for Quality in Health Care*. 2007. Volume 19, Number 6: pp. 349 – 357

No. Item	Guide questions/description	Reported on Page #
**Domain 1: Research team and reflexivity**		
Personal Characteristics		
1. Inter viewer/facilitator	Which author/s conducted the interview or focus group?	ST, AC, AF and ZL.
2. Credentials	What were the researcher's credentials? E.g. PhD, MD	ST - PhD<br>AC – MPH<br>AF - MPH<br>ZL – BSc
3. Occupation	What was their occupation at the time of the study?	ST – PhD candidate at SSHSPH, NUS<br>AC – MPH student, SSHSPH, NUS<br>AF and ZL – Research Assistant, SSHSPH, NUS
4. Gender	Was the researcher male or female?	AT, AC, AF and ZL: Female
5. Experience and training	What experience or training did the researcher have?	ST is experienced qualitative researcher with prior experience across different qualitative projects.<br>AC had basic training in qualitative research (MPH).<br>AF and ZL were new to qualitative research.<br>ST led qualitative methods and software training for rest. ST supervised early interviews.
Relationship with participants		
6. Relationship established	Was a relationship established prior to study commencement?	No prior relationship between the interviewers and interviewees existed.
7. Participant knowledge of the interviewer	What did the participants know about the researcher? e.g. personal goals, reasons for doing the research	Potential participants were informed about the study and its purpose and those agreeable were enrolled for interviewing.

		They were further informed about the study by interviewers with sharing of study information sheet (approved by the Institutional Ethics Review Board).
8. Interviewer characteristics	What characteristics were reported about the inter viewer/facilitator? e.g. Bias, assumptions, reasons and interests in the research topic	Participants knew about the work scope of the interviewers.
**Domain 2: study design**		
Theoretical framework		
9. Methodological orientation and Theory	What methodological orientation was stated to underpin the study? e.g. grounded theory, discourse analysis, ethnography, phenomenology, content analysis	The study was based on the principles of qualitative description, in which qualitative approach is adopted to get participants' experiences and perceptions of the phenomenon being studied (post-stroke healthcare seeking experience of stroke survivors and their caregivers).<br><br>Further researchers used thematic analysis to analyze the data.<br>Please refer Page – 6 for further details on data analysis.
Participant selection		
10. Sampling	How were participants selected? e.g. purposive, convenience, consecutive, snowball	Purposive sampling was used.
11. Method of approach	How were participants approached? e.g. face-to-face, telephone, mail, email	Participants were approached face-to-face in outpatient rehabilitation setting and outpatient clinic setting, while email was sent for the support organization for stroke survivors and their caregivers.
12. Sample size	How many participants were in the study?	61 total participants: 35 caregivers and 26 stroke survivors.
13. Non-participation	How many people refused to participate or dropped out? Reasons?	Not captured.
Setting		
14. Setting of data collection	Where was the data collected? e.g. home, clinic, workplace	Participants were either interviewed at their homes, or outpatient clinic setting or in a community setting of participant preference.

15. Presence of non-participants	Was anyone else present besides the participants and researchers?	Non-participants were not present when interviews were conducted.
16. Description of sample	What are the important characteristics of the sample? e.g. demographic data, date	Information about the demographic information was collected for all participants and is presented in **Table 3**.
Data collection		
17. Interview guide	Were questions, prompts, guides provided by the authors? Was it pilot tested?	Interview guide was used to collect data and it was pilot tested.<br>Topic guide is presented in **Table 1**.
18. Repeat interviews	Were repeat inter views carried out? If yes, how many?	There were no repeat interviews.
19. Audio/visual recording	Did the research use audio or visual recording to collect the data?	Interviews were audio-recorded and transcribed verbatim.
20. Field notes	Were field notes made during and/or after the inter view or focus group?	Field notes were taken, and memos were written post interviews.
21. Duration	What was the duration of the inter views or focus group?	Interviews generally lasted from 28 to 58 minutes.
22. Data saturation	Was data saturation discussed?	Yes, data collection was stopped once the researchers observed recurring themes emerging.
23. Transcripts returned	Were transcripts returned to participants for comment and/or correction?	Transcripts were not returned to the interviewees.
**Domain 3: analysis and findings**		
Data analysis		
24. Number of data coders	How many data coders coded the data?	ST was the primary coder, with a subset of interviews being coded by AC. Coding consistency was checked by coders via discussions among the team.
25. Description of the coding tree	Did authors provide a description of the coding tree?	The authors provided a description of main themes and sub-themes along with providing a diagrammatic summary based on synthesis of themes.
26. Derivation of themes	Were themes identified in advance or derived from the data?	Themes were related to subject matter in the topic guide, along with

		emerging themes which were inductively derived from data.
27. Software	What software, if applicable, was used to manage the data?	NVivo 12 qualitative data analysis software.
28. Participant checking	Did participants provide feedback on the findings?	Researchers didn't conduct participant checking of research findings.
Reporting		
29. Quotations presented	Were participant quotations presented to illustrate the themes/findings? Was each quotation identified? e.g. participant number	Yes, quotations were identified by participant number and whether the participant was a patient or caregiver.
30. Data and findings consistent	Was there consistency between the data presented and the findings?	We have attempted to present our findings in a clear manner, consistent with the data collected.
31. Clarity of major themes	Were major themes clearly presented in the findings?	Yes
32. Clarity of minor themes	Is there a description of diverse cases or discussion of minor themes?	Yes, the authors have presented description of all major and minor themes within the manuscript
Abbreviations: ST = Shilpa Tyagi, AC= Audrey Swee Ling Chan, AF= Aysha Farwin, ZL=Zunairah Binti Lukman, SSHSPH, NUS = Saw Swee Hock School of Public Health, National University of Singapore.		
