# Supplementary material for: Seeking healthcare services post-stroke: a qualitative descriptive study exploring family caregiver and stroke survivor perspectives in an asian setting
Source: BMC Neurol. 2021 Nov 5;21:429. doi: 10.1186/s12883-021-02463-7 (PMC8569985; doi:10.1186/s12883-021-02463-7)
Supplement: Supplementary file 2 — Additional file 2. [file 12883_2021_2463_MOESM2_ESM.docx]

**Seeking healthcare services post-stroke: a qualitative descriptive study exploring family caregiver and stroke survivor perspectives in an Asian setting.**

Shilpa TYAGI^1^, Nan LUO^1^, Chuen Seng TAN^1^, Kelvin Bryan TAN^2^, Boon Yeow TAN^3^, Edward MENON^4^, N. Venketasubramanian^5,6^, Wei Chin LOH^4^, Shu Hui FAN^4^, Kenneth Lam Thuan YANG^3^, Audrey Swee Ling CHAN^1^, Aysha FARWIN^1^, Zunairah Binti LUKMAN^1^, Gerald Choon-Huat KOH^1*^

^1^Saw Swee Hock School of Public Health, National University of Singapore, Singapore

^2^Policy Research & Economics Office, Ministry of Health, Singapore

^3^St. Luke's Hospital, Singapore

^4^St. Andrew’s Community Hospital, Singapore

^5^Raffles Neuroscience Centre, Raffles Hospital, Singapore

^6^Singapore National Stroke Association, Singapore

***Corresponding Author**

Dr Gerald Choon-Huat Koh

Saw Swee Hock School of Public Health, National University of Singapore

12 Science Drive 2, #10-01

Singapore 117549

Tel (65) 6516 4979, Fax (65) 6779 1489

Email: [gerald_koh@nuhs.edu.sg](mailto:gerald_koh@nuhs.edu.sg)

**Interview guide for caregivers and stroke survivors**

| **CAREGIVER** |
| --- |
| - Ever since (stroke patient’s name) has had stroke, which are the places where you have gone to seek care for the stroke patient (like acute hospital, community hospital, polyclinics or public primary care clinic, general practitioner led private primary care clinic, specialist outpatient clinic, or traditional Chinese medicine, etc.)? - For each service the participant lists, ask the following:   - Can you tell me more about the last time you went to (insert healthcare setting)?   - Why did you go there? (*Probe about perceived need*) - What are some of the common reasons (stroke patient’s name) sought healthcare after the stroke? - When did you recently think about seeking healthcare for (stroke patient’s name) but decided not to go? Can you tell me more about the situation? - Could you describe one good experience seeking healthcare? - Could you describe one bad experience seeking healthcare? - How do you decide between different healthcare settings, which one to go to? - Who makes the final decision? (*Probe: Do family members have a say?*) - Is there anything you would like to add that we have not covered? |
| **STROKE SURVIVOR** |
| - Ever since you had stroke, which are the places where you have gone to seek care (like acute hospital, community hospital, polyclinics or public primary care clinic, general practitioner led private primary care clinic, specialist outpatient clinic, or traditional Chinese medicine, etc.)? - For each service the participant lists, ask the following:   - Can you tell me more about the last time you went to (insert healthcare setting)?   - Why did you go there? (*Probe about perceived need*) - What are some of the common reasons you sought healthcare after the stroke? - When did you recently think about seeking healthcare but decided not to go? Can you tell me more about the situation? - Could you describe one good experience seeking healthcare? - Could you describe one bad experience seeking healthcare? - How do you decide between different healthcare settings, which one to go to? - Who makes the final decision? (*Probe: Do family members have a say?*) - Is there anything you would like to add that we have not covered? |
